# The Role of Lake Expansion in Altering the Wetland Landscape of the Prairie Pothole Region, United States

**DOI:** 10.1007/s13157-015-0728-1

**Published:** 2015-12-30

**Authors:** Melanie K. Vanderhoof, Laurie C. Alexander

**Affiliations:** 1 ORISE c.o. U.S. EPA Office of Research and Development, National Center for Environmental Assessment, 1200 Pennsylvania Ave. NW (8623-P), Washington, DC 20460 USA; 2 Present Address: U.S. Geological Survey, Geosciences and Environmental Change Science Center, DFC, MS980, P.O. Box 25046, Lakewood, CO 80225 USA

**Keywords:** Lakes, Surface water, Connectivity, Prairie pothole region, Depressional wetlands, Wetland loss, Landsat, Climate

## Abstract

Interannual variation in lake extent is well documented in the Prairie Pothole Region, but the role of surface-water expansion, including lake expansion, in merging with and subsuming wetlands across the landscape has been minimally considered. We examined how the expansion of surface-water extent, in particular, the expansion of lakes across parts of the Prairie Pothole Region can alter landscape-level hydrologic connectivity among substantial numbers of previously surficially disconnected wetlands. Temporally static wetland, lake, and stream datasets were fused with temporally varying Landsat-derived surface-water extent maps (1990–2011) to quantify changes in surface-water connectivity. Under deluge conditions, lakes were found to create significantly larger complexes of surficially-connected wetlands relative to non-lake surface-water connections (e.g., only wetlands or wetlands and streams). Analysis of three specific lakes showed that lakes can merge with and subsume wetlands located kilometers to tens of kilometers from the National Wetland Inventory defined lake perimeter. As climate across the Prairie Pothole Region is highly variable, understanding historic patterns of surface-water expansion and contraction under drought-to-deluge conditions will be integral to predicting future effects of climate change on wetland function, loss and influence on other aquatic systems, including downstream waters.

## Introduction

The Prairie Pothole Region (PPR) in central North America is known for its high density of depressional wetlands and lakes, a relic of multiple glacial advances and retreats (Flint 1971). These wetlands and lakes provide habitat for large populations of waterfowl (Sorenson et al. 1998). The region is also characterized by substantial spatiotemporal variability in air temperature and precipitation (Bryson and Hare 1974), to which wetland and lake water-levels are highly responsive (LaBaugh et al. 1998; Winter and Rosenberry 1998; Johnson et al. 2004; Liu and Schwartz 2011). Water levels of Devils Lake, North Dakota, for example, have been measured since 1867 and are considered to be a regional indicator of long-term hydrological conditions (LaBaugh et al. 1996; Wiche 1996). Change in the areal extent of PPR wetlands and lakes is driven predominately by seasonal and multi-year patterns in precipitation (Zhang et al. 2009), whereas spatial variability in the response to climate is attributed to topography (Rover et al. 2011), as well as to variability in groundwater interactions (LaBaugh et al. 1996). Although changes in surface-water extent in the PPR have primarily been evaluated at specific sites, variability in surface-water extent has also been mapped at a landscape scale (Kahara et al. 2009; Niemuth et al. 2010; Vanderhoof et al. 2015).

Variation in surface-water extent is important, not only for quantifying change to waterfowl habitat, but also for examining how surface-water expansion can create connections between previously disconnected features and, as water levels rise, result in the temporary loss of wetland function. Surface-water connections between water features (e.g., wetlands, lakes streams) is relevant as federal protection of wetlands and wetland functions under the Clean Water Act and Clean Water Rule considers connectivity and effects of wetlands with respect to down-stream waters (U.S. EPA and U.S. ACE 2015). For most wetlands in the PPR, the primary mechanism of water gain is precipitation and water loss is evapotranspiration (Winter and Rosenberry 1998). However, under wet conditions, as wetland storage capacity is exceeded, many PPR wetlands may intermittently connect or contribute water to other wetlands, lakes and streams through temporary overland or shallow groundwater flows, wetland “fill and spill” mechanisms, wetlands merging in low-relief areas and/or ephemeral channels (Rains et al. 2008; Cook and Hauer 2007; Sass and Creed 2008; Kahara et al. 2009; Philips et al. 2011; Wilcox et al. 2011; Shaw et al. 2012). Wetlands can also become subsumed by streams, lakes or other wetlands in low-relief areas during flood events or wet periods, resulting in the temporary loss of wetland function until water levels recede (Junk et al. 1989; Galat et al. 1998; Mortsch 1998). Many of these connections can be detected remotely (e.g., Rover et al. 2011). However, relatively few studies have examined how interannual variability in surface-water extent affects the abundance of surface-water connections among water features within the PPR (Leibowitz and Vining 2003; Shaw et al. 2013; Vanderhoof et al. 2015).

This study examines how surface-water expansion, and in particular, lake expansion, acts to alter the wetland landscape. A Landsat time series (1990–2011) was used to document variation in surface-water extent over time and was related to wetland, lake and stream datasets. Our objectives were to quantify 1) the number of surface-water connections among wetlands, lakes and streams, and the size of surface connected wetland complexes, created by deluge conditions; 2) the areal extent of lake expansion from drought to deluge conditions; and 3) the number of wetlands that merge with or are subsumed by three specific lakes as a result of expansion. Long-term trends in climate could have implications for the distribution, persistence and movement of surface-water, and the gain or loss of wetland habitat and function across the region (Millett et al. 2009; Johnson et al. 2010).

## Methods

### Study Area

**Fig. 1:**
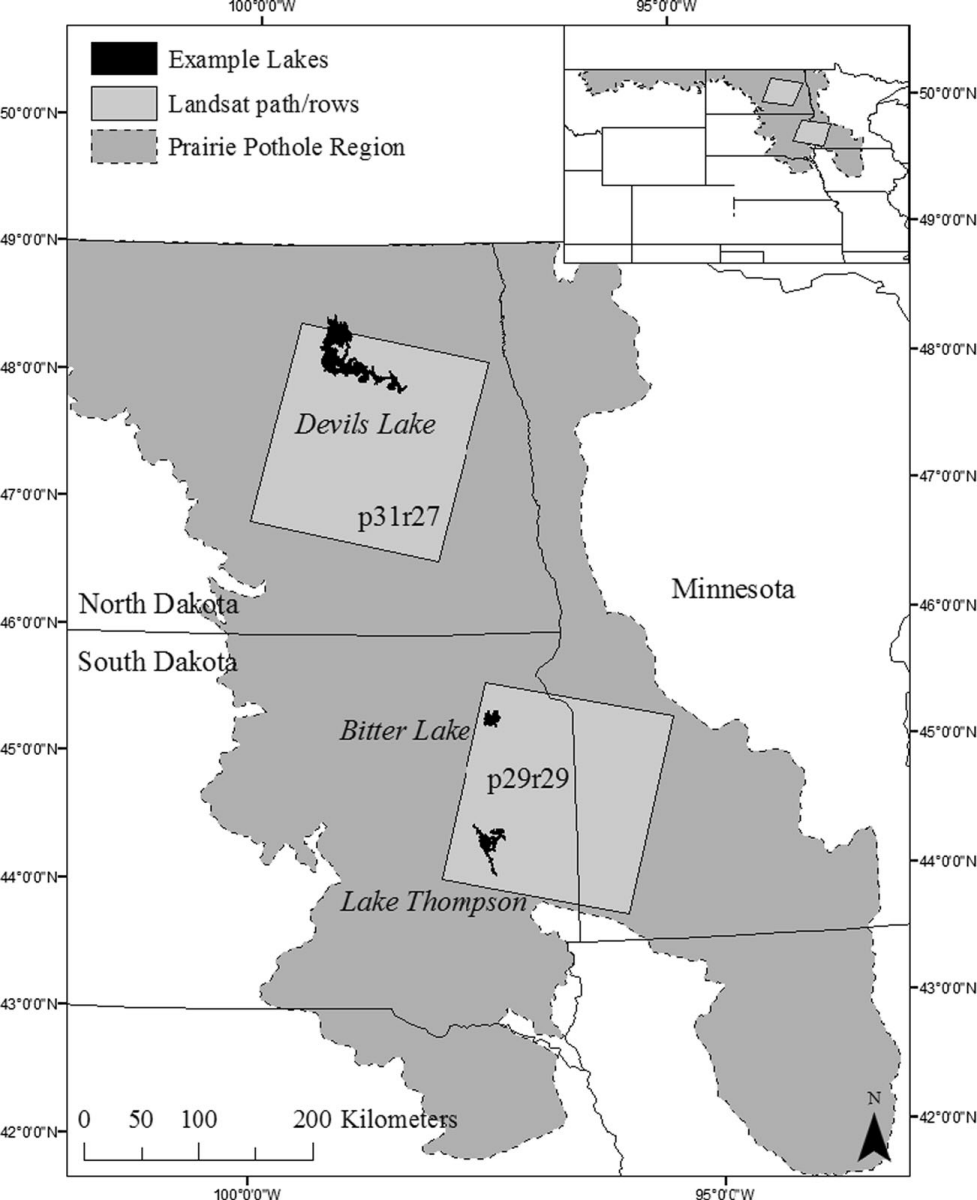
Location of study sites and three specific lakes (i.e., Devils Lake, Bitter Lake, Lake Thompson) analyzed within the Prairie Pothole Region of the United States. The study area for the lake-scale analysis was defined as the maximum lake extent observed over the time series (shown). Landscape scale analysis was restricted to the two Landsat path/rows shown, while a third Landsat path/row time series (p31/r26, not shown) was used to map the northern extent of the maximum Devils Lake extent

The landscape-scale study area consists of two non-adjacent Landsat path/rows (p29/r29 and p31/r27) within the United States portion of the PPR (Fig. 1). Landcover across both path/rows is dominated by cultivated crops (51 and 62 % for p31/r27 and p29/r29, respectively), hay/pasture (16 and 10 % for p31/r27 and p29/r29, respectively), and herbaceous vegetation (14 and 13 % for p31/r27 and p29/r29, respectively), as determined from the 2011 National Land Cover Database (Homer et al. 2015). Summer (June–August) mean daily temperatures were similar for p31/r27 and p29/r29 (19.9 and 20.9 °C, respectively), as were winter (December–February) mean daily temperatures (−9.2 and −8 °C, respectively) from 1981 to 2010. However, mean annual precipitation was lower for p31/r27 (496 mm yr^−1^) than for p29/r29 (649 mm yr^−1^) (NOAA NCDC 2014). Three specific lakes were selected for further analysis within the study area, 1) Bitter Lake (1358 ha) and 2) Lake Thompson (4135 ha), both in the Prairie Coteau, South Dakota, and 3) Devils Lake (19,908 ha) in the Drift Plains in North Dakota. Lake sizes were measured from the National Wetland Inventory (NWI) database, which was primarily mapped in 1979–1984 for our study area (USFWS 2010). These lakes were not intended to be representative of the PPR but were selected because they showed highly variable lake extent between dry and wet conditions within the 1990–2011 time period of our selected Landsat observations.

Bitter Lake occurs within a north–south chain of lakes in the Prairie Coteau, formed where there was minimal ice shear with glacial retreat (USGS 2013a). Lake Thompson occurs along the East Fork of the Vermillion River. Under deluge conditions, the lake merges with Lake Henry to the north, as well as Lakes Whitewood and Preston to the northeast. Devils Lake occurs in a closed basin. Under deluge conditions, Devils Lake merges with Lake Irvine, Lake Alice and Dry Lake, all to the north, as well as Stump Lake to the southeast. Above 444 msl, the lake flows into the Sheyenne River, although this spill has not occurred in approximately 1000 years (USGS 2013a). Under deluge conditions, the extent of Devils Lake extends outside of p31/r27, therefore a third path/row (p31/r26) was also processed but used only to include the full extent of Devils Lake.

### Image Processing

**Fig. 2:**
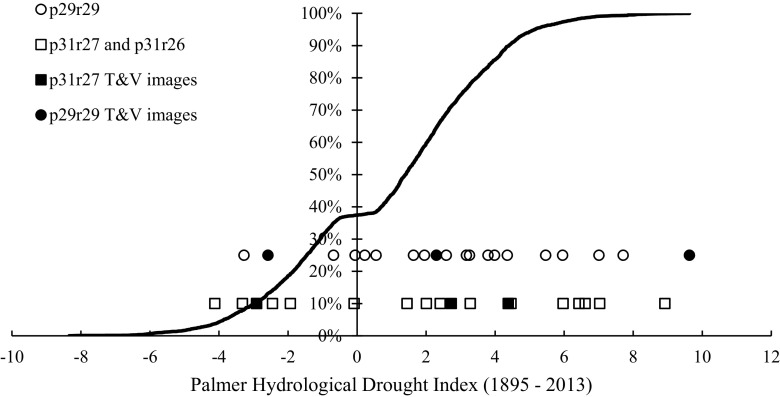
The Cumulative Distribution Function (CDF) for monthly Palmer Hydrological Drought Index (PHDI) values from 1895 to 2013 averaged across the NOAA NCDC Division 5 in North Dakota, Division 7 in South Dakota and Division 7 in Minnesota (March to November) (NOAA NCDC 2014). The distribution of PHDI values for the time series of Landsat images is also shown (via *circles* and *squares*), with the images utilized for the threshold and validation analysis (T&V) indicated

Seventeen Landsat images for p29/r29 and sixteen Landsat images for p31/r27 with <10 % cloud cover were selected to coincide with snow-free conditions (Table 1). The time series (1990–2011) represented a wide range of interannual hydrological conditions (Fig. 2). The images were atmospherically corrected and converted to surface reflectance values using the Landsat Ecosystem Disturbance Adaptive Processing System (LEDAPS; Masek et al. 2006). Surface-water was identified using 1) the Matched Filtering algorithm in the ENVI software package (Exelis Visual Information Solutions, Inc, Herndon, VA) and 2) a threshold analysis. The Matched Filtering algorithm is designed to detect the abundance of a known endmember (e.g., water) against a composite of unknown background endmembers (e.g., vegetation, soil) using a partial unmixing technique (Turin 1960; Frohn et al. 2012). A minimum noise fraction transformation was applied to the Matched Filtering outputs to reduce noise in the data (Green et al. 1988). The output values were then linearly stretched to enhance the data by maximizing the spread of pixel values. Lastly, impervious surfaces, defined using a landcover layer (Homer et al. 2015), were masked out to reduce errors of commission.

**Table 1:** Landsat TM images utilized in the analysis

Path/row	Landsat TM image	Path/row	Landsat TM image
p29/r29	10-May-90	p31/r27 and p31/r26	9-Jun-90
p29/r29	13-May-91	p31/r27 and p31/r26	12-Jun-91
p29/r29	15-May-92	p31/r27	27-Apr-92
p29/r29	23-Sep-93	p31/r26	5-May-92
p29/r29	15-Oct-95	p31/r27 and p31/r26	26-Oct-94
p29/r29	14-Jun-97	p31/r27 and p31/r26	27-Sep-95
p29/r29	30-Apr-98	p31/r27 and p31/r26	14-Jul-97
p29/r29	*8-May-01	p31/r27 and p31/r26	1-May-99
p29/r29	19-Nov-02	p31/r27 and p31/r26	9-Jul-01
p29/r29	28-Apr-03	p31/r27 and p31/r26	5-Oct-04
p29/r29	1-Apr-05	p31/r27 and p31/r26	*18-Jun-05
p29/r29	4-Apr-06	p31/r26	15-Aug-06
p29/r29	13-Oct-06	p31/r27	9-Sep-06
p29/r29	15-Apr-10	p31/r27 and p31/r26	12-Sep-07
p29/r29	8-Oct-10	p31/r27 and p31/r26	1-Sep-09
p29/r29	*5-Jun-11	p31/r27 and p31/r26	6-Oct-10
p29/r29	11-Oct-11	p31/r27 and p31/r26	*5-Jul-11
		p31/r27 and p31/r26	11-Sep-11

The time series within p31/r26 was used only to map the northern extent of Devils Lake. Data from similar dates were combined for p31/r27 and p31/r26 where necessary (1992 and 2006). Landscape analysis was limited to shared years between p29/r29 and p31/r27. Dates defined as deluge are starred

The algorithm output maps representing the fractional water per pixel were classified into two versions of wet/dry maps using a threshold analysis, inundated (Inun) and non-inundated, and saturated (Sat) (representing saturated and inundated) and non-saturated. The per pixel fraction water was derived for 1108 points representing diverse cover classes including inundated soil (i.e., open water) (258 points), saturated soil (i.e., visibly wet soil adjacent to open water) (239 points), wet channels and swales (261 points), non-photosynthetic vegetation (167 points), and upland photosynthetic vegetation (183 points). These points were randomly selected and distributed across the two Landsat path/rows for three dates per Landsat path/row (30 April 2004, 13 October 2006 and 8 October 2010 for p29/r29; 1 July 2004, 5 October 2004 and 9 September 2006 for p31/r27). The cover category was classified using 1 m resolution National Agricultural Imagery Program (NAIP) imagery. The inundated cover class (threshold ≥ 0.53) showed the highest mean fraction water and represents a high confidence of surface-water. This cover class was distinguished from saturated (threshold ≥ 0.26), which showed the second highest mean fraction water. The lower threshold of the saturated class resulted in this class including more mixed pixels (e.g., shallow water or shallow sub-surface flow, wetland edges and vegetated water) (e.g., Sass and Creed 2008), relative to the inundated cover class. The saturated class was distinguished from upland photosynthetic vegetation, which showed a higher mean fraction water relative to non-photosynthetic vegetation. Most small (i.e., ~3–10 m wide) channels and swales represented a minor fraction of individual Landsat pixels and were spectrally indistinguishable from non-saturated cover types. Differences in the fraction water were larger between cover classes than between dates, so cover class thresholds were applied across all dates and both path/rows. The outputs were inundation and saturation cover maps over the time series.

### Validation Analysis

The inundated and saturated cover maps were validated using a random point analysis of 1500 points. A total of 250 independent points per path/row were used, derived from the same NAIP dates as used for the threshold analysis, due to limited overlap between Landsat images and NAIP collection dates. Surface-water versus upland classifications were compared against NAIP classified upland, saturated and inundated points. Upland was defined as any pixel that did not meet the inundated, for the inundated validation, or saturated, for the saturated validation, threshold of fraction water; therefore NAIP pixels classified as saturated were considered upland in the inundation validation and surface water in the saturation validation. In this analysis, producer’s accuracy was the probability that the Landsat pixel was classified as surface water, given surface water was present in the NAIP imagery; user’s accuracy was the probability that surface water was present in the NAIP imagery, given a Landsat pixel classified as surface water. The overall accuracy of our classification was 96.5 % for the saturated and 95.3 % for the inundated surface-water maps. The producer’s accuracy for wetlands was 94.6 % for the saturated and 77.9 % for the inundated maps, while the user’s accuracy for wetlands was 88.4 % for the saturated and 98.3 % for the inundated maps (Table 2). The lower producer’s accuracy for the inundated surface-water maps was a consequence of using a conservative threshold, which excluded more mixed pixels. The saturated threshold, in contrast, allowed more mixed pixels or small wetlands, to be identified. Consistent with this, the relative bias (rb) for the saturated threshold showed an over-estimation of surface-water extent (rb = 0.1), while the inundated threshold showed an under-estimation of surface-water extent (rb = −0.3). The Dice coefficient was also provided to indicate the degree of overlap or agreement between the Landsat and NAIP imagery (Table 2) (Fleiss 1981). We note that from this point forward, areas classified as inundated or saturated will be referred to as surface waters, whereas reference to lakes or wetlands will be defined as subsets of polygons in the NWI dataset (USFWS 2010), as explained in “Additional Datasets.”

**Table 2:** Accuracy assessment for surface-water extent maps, comparing Landsat derived surface-water (per pixel fraction water above inundation or saturation threshold) and upland (per pixel fraction water below inundation or saturation threshold) classification maps, to 1 m NAIP aerial imagery

Saturation map accuracy	NAIP – wetland	NAIP – upland	Total points
Landsat – wetland	283	37	320
Landsat – upland	16	1164	1180
Total	299	1201	1500
Producer accuracy for wetland (%)	94.6		
User accuracy for wetland (%)	88.4		
Overall accuracy (%)	96.5		
Dice coefficient	0.9		
Relative bias	0.1		
Inundation map accuracy	NAIP – wetland	NAIP – upland	Total points
Landsat – wetland	232	4	236
Landsat – upland	66	1198	1264
Total	298	1202	1500
Producer accuracy for wetland (%)	77.9		
User accuracy for wetland (%)	98.3		
Overall accuracy (%)	95.3		
Dice coefficient	0.8		
Relative bias	−0.3		

### Additional Datasets

The NWI dataset (USFWS 2010) was used as a static, or non-changing, reference layer against which comparisons of interannual variation in surface-water extent could be made. The NWI dataset was designed to represent wetland extent under “average” hydrological conditions (USFWS 2010). Wetland boundaries that were internal to a single, continuous NWI wetland were dissolved to ensure that connectivity was classified similarly for different parts of a continuous wetland. However this step also meant that connectivity was classified corresponding to the wetland’s most permanent hydroperiod. Lakes were defined as NWI polygons classified as lacustrine (Cowardin et al. 1979). We note that as conditions such as water depth and vegetation dynamics change over time, the corresponding nomenclature (e.g., wetland, lake) can also change (Bjӧrk 2010), especially during recent years (e.g., 1994–2011) which represent a particularly wet phase in the climate of the PPR (Mushet et al. 2015). Stream occurrence was defined by the high resolution NHD (1:24,000) (USGS 2013b). The NHD was revised to only include streamlines showing a downstream connection (85 % of total high resolution NHD stream length within the study area). A stream buffer was applied to account for the nationally reported digital inaccuracy (±14 m) in the lateral location of stream features (USGS 2000). Stream and river polygons designated by the NHD Area layer were merged with the buffered stream layer.

### Landscape and Lake-Scale Analysis

“Surface water connection” is used as a general term indicating multiple mechanisms, including wetland fill-and-spill, merging and subsuming of wetlands by lakes or other wetlands and stream overbank flow. In using this term we make no assumption about shifts or loss of wetland function. Instead, the Landsat thresholds are used to distinguish between wetland loss through being subsumed, and wetlands spilling or merging with other water features. Wetlands showing a surface-water connection to a lake using both the saturated and inundated thresholds were assumed to have been subsumed, i.e., to have become part of the lake. Wetlands connected using the saturated threshold, but not the inundated threshold, were assumed to have merged with the lake, but to retain wetland functions (i.e., were not subsumed). Although wetland functions can be temporarily lost by a wetland being subsumed by a lake, or shifted when a wetland is subsumed by another wetland, prior wetland function can be expected to resume if water levels subside and wetlands disconnect (Mortsch 1998).

Surface-water connections were quantified by relating the Landsat-derived surface-water extent to the wetland, lake and stream datasets. Wetlands and lakes were considered to show a surface-water connection with a stream if they intersected a buffered stream polygon, or intersected a Landsat-derived surface-water polygon that in turn, intersected a buffered stream polygon. Wetland–lake and wetland–wetland surface-water connections were identified when multiple features co-occurred within a single Landsat-derived surface-water polygon. Any wetland that did not intersect a buffered stream polygon or co-occur in a continuous surface-water polygon with a lake or other wetland was considered to show no observed connection. This classification does not mean that hydrological connections with other features are absent, just that they were not identified using our approach.

**Fig. 3:**
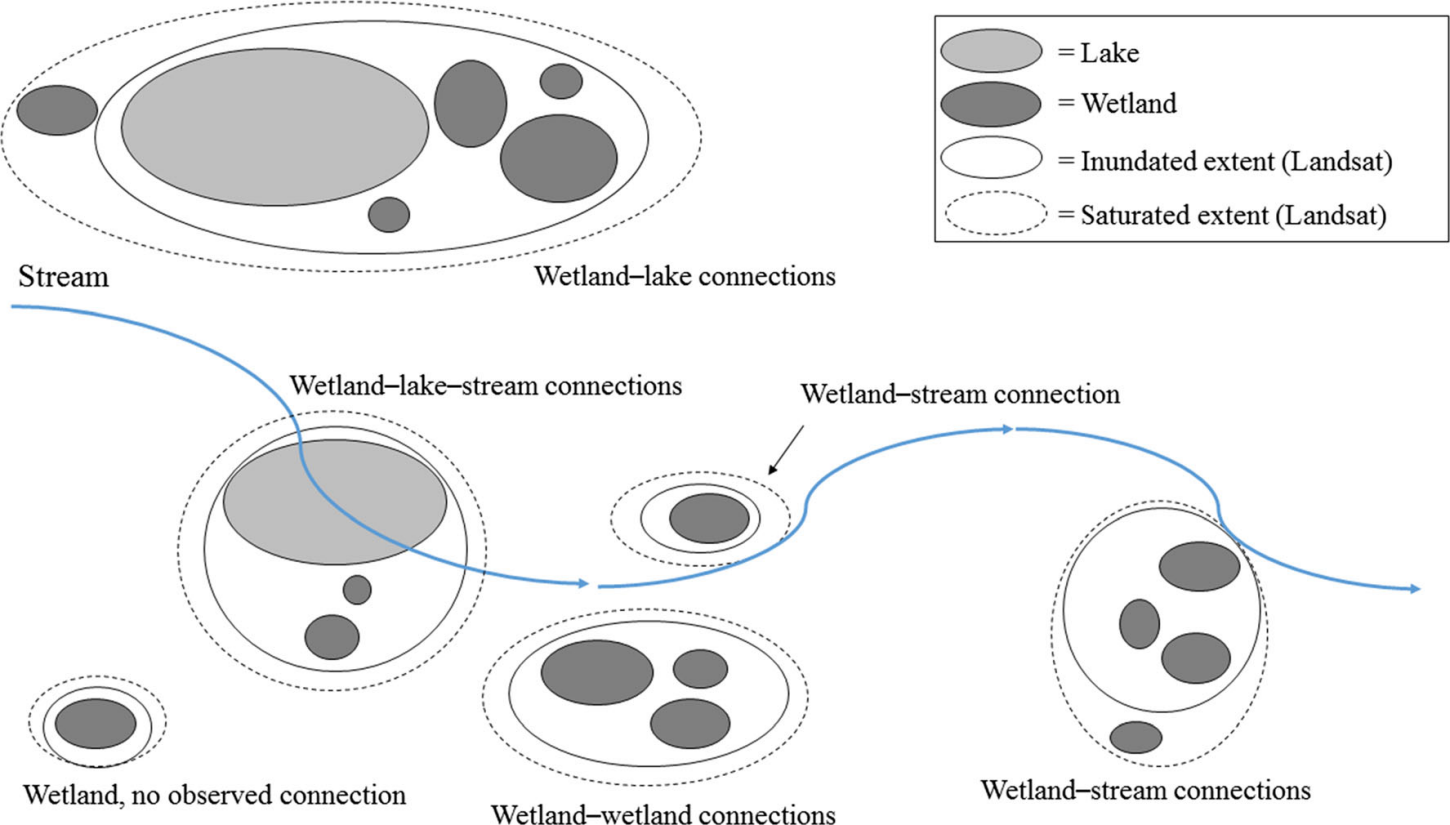
A simplified schematic showing the different types of surface-water connections identified in the landscape-scale analysis. Differences in connections between inundated and saturated thresholds are interpreted as differences in wetlands subsumed (connected using inundated extent) versus merged (connected only under the saturated extent)

The number of surface-water connections between water features increased greatly under very wet conditions (Vanderhoof et al. 2015). Therefore, the abundance of surface-water connections and wetland–lake–stream complex sizes were quantified in deluge conditions, defined as the two images in the time series for each Landsat path/row (p29/r29 – 8 May 2001 and 5 June 2011; p31/r27 – 18 June 2005 and 5 July 2011) with the largest total wet (saturated) area (Table 1). Complex size was calculated as the number of features showing surficial connections, or co-occurring in a single, continuous Landsat-derived surface-water polygon, and included wetlands, lakes and streams as relevant (Fig. 3). For each of the three specific lakes analyzed, the study area was defined as the maximum lake extent.

Landsat imagery provides global data coverage at regular return intervals, but due to the 30 m pixel spatial resolution, analysis of surface-water connections can be biased toward detecting only those that occur through the expansion of relatively broad features. Intermittent or temporary linear connections (e.g., ephemeral channels, swales, ditches, fill-and-spill events) that connect some waters (Shaw et al. 2012) are difficult to detect with Landsat and often are not well documented by NHD, which has been shown to inconsistently map such features (Lang et al. 2012; Fritz et al. 2013). Although finer spatial resolution imagery may expand the types of connectivity captured, these data sources are often collected on-demand, reducing our ability to capture a wide range of climate conditions. Our approach documents an incomplete estimate of total surface-water connections but is suitable for the aim of this analysis, which was to explore the role of lake expansion in producing surface-water connections with and subsuming wetlands.

## Results

### Surface-Water Expansion

**Fig. 4:**
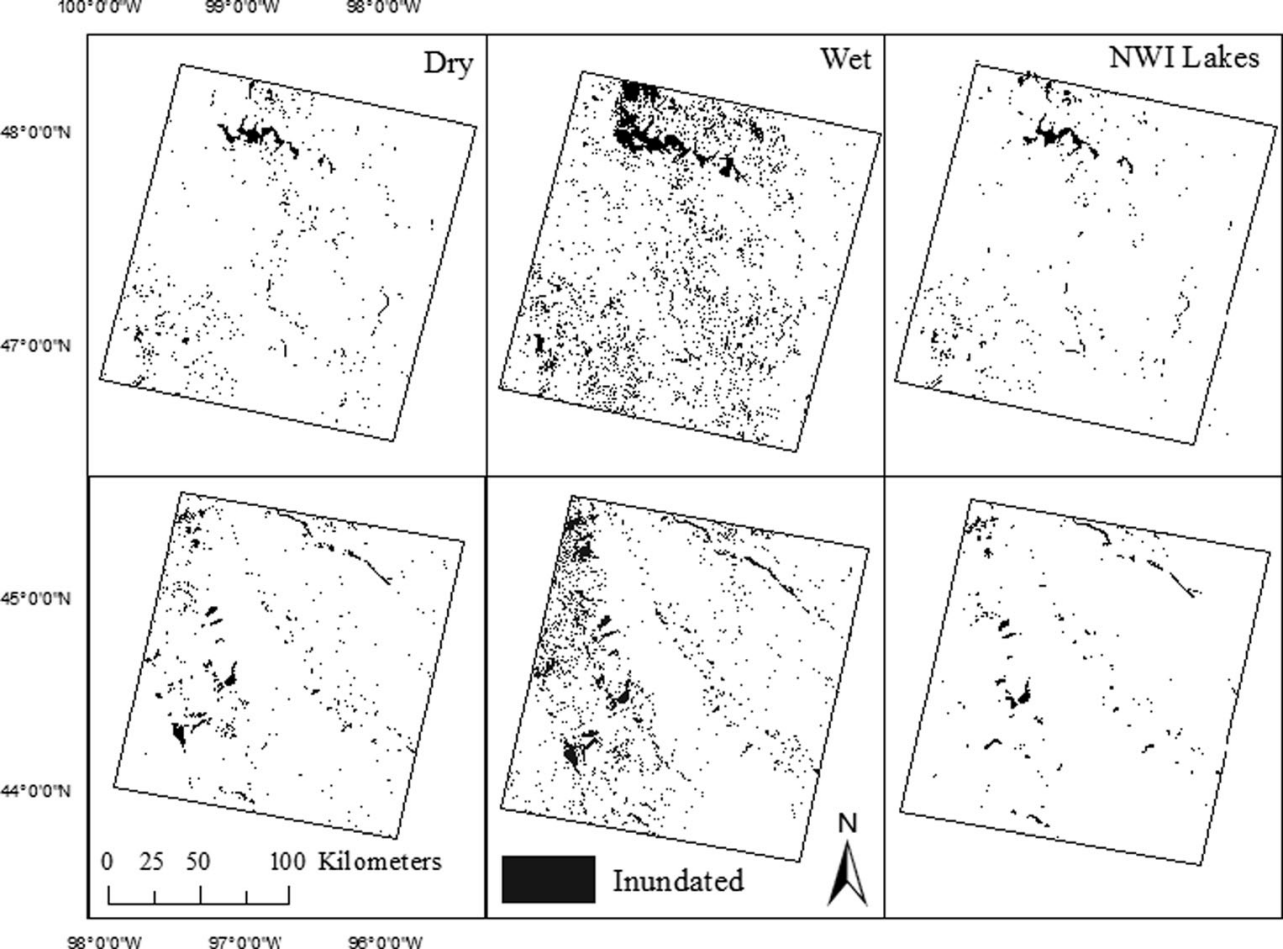
Patterns of inundation for dry, or *Pr*(0.06) cumulative distribution function (CDF) of the Palmer Hydrological Drought Index (PHDI) (spring 1990) (*left*) and wet, or *P*r(0.99) CDF PHDI (spring 2011) (*middle*) conditions for p31/r27 (*top row*) and p29/r29 (*bottom row*). Lake extent as defined by the National Wetland Inventory (NWI) dataset (right). NOTE: Not all wetlands are visible due to the scale of the images

**Fig. 5:**
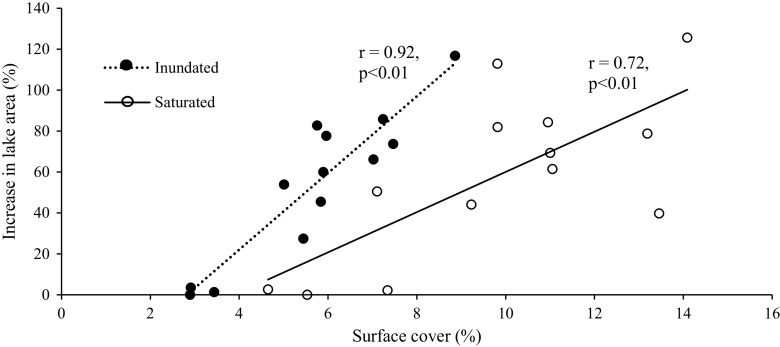
Increase in lake area (relative to the smallest Landsat-derived lake extent) across two Landsat path/rows (p29/r29 and p31/r27)

The variability in total surface-water extent for both path/rows was substantial from the driest to the wettest date in the time series, increasing 206 and 203 % for inundated and saturated thresholds, respectively. Total surface-water extent fluctuated over the time series with the driest date occurring in spring/early summer 1990 and the wettest date occurring in early summer 2011. This represented an increase from 2.9 to 8.9 % inundated cover, and 4.7 to 14.1 % saturated cover (Fig. 4). By count, NWI-defined lakes comprised a very small percent of all NWI features (0.4 %) but a much larger portion by area (21 % of total NWI area). Further, 15.9 % (53.2 % by area) of the lakes showed outflow to downstream connected streams. Landsat surface-water classified as lake varied substantially across the time series, increasing 117 and 126 % for the inundated and saturated thresholds respectively (Fig. 5). Saturation was found to be substantial at lake edges with the saturated threshold, relative to the inundated threshold, producing larger lake extents between drought and deluge conditions (Fig. 5).

Although “no observed connection” was the dominant observed state for wetlands across the study area, of the observed surface-water connections in deluge conditions, lakes played a role in 10 % of all wetland surface-water connections and 22 % of stream-connected connections (Table 3). Further, under deluge conditions, lakes were found to create significantly larger complexes of surficially-connected wetlands, relative to non-lake surface-water connections (e.g., wetlands only, wetlands–streams; Table 3). For example, stream-connected complexes containing a lake contained almost 24 times the number of wetlands on average, relative to non-lake stream-connected complexes (Table 3). Most wetlands showing surface-water connections with lakes, streams or other wetlands were found to be subsumed (60 to 88 % depending on complex type) relative to merging with the complex (12 to 40 % depending on complex type) (Table 3).

**Table 3:** The relative frequency and mean complex size by wetland connection type under saturated, deluge conditions

Wetland connection type	Relative percent	Relative percent (stream connected)	Complex size (subsumed wetlands)	Complex size (subsumed and merged wetlands)
Lake complexes				
Wetland–lake connection (no stream or stream not downstream-connected)^1^	4.7		7.3 ± 0.9^b^	11.2 ± 1.1^b^
Wetland–lake–stream connection^1^	4.8	22.5	21.4 ± 8.5^a^	35.6 ± 14.9^a^
Non-lake complexes				
Wetland–wetland connection (no stream or stream not downstream-connected)^2^	73.8		2.9 ± 0.03^c^	3.3 ± 0.02^c^
Wetland–stream connection^1^	16.6	77.5	1.2 ± 0.01^d^	1.5 ± 0.02^d^
Wetlands, no observed connection	72.1			

The relative percent for no observed connection is a function of all National Wetland Inventory (NWI) wetlands, while the relative percent for connections are shown as function of all wetland connection types under deluge conditions. Streams refer to a subset of downstream-connected stream lines. Mean complex size, plus and minus standard error shows the number of wetlands in a single, surficially-connected complex under deluge conditions. Significant differences (*p* < 0.05, superscripts) were derived using ANOVA and Tukey HSD post-hoc tests

^1^Only one wetland required for a connection; ^2^Two wetlands required for a connection as most contain only wetlands

### Lake-Scale Analysis

**Fig. 6:**
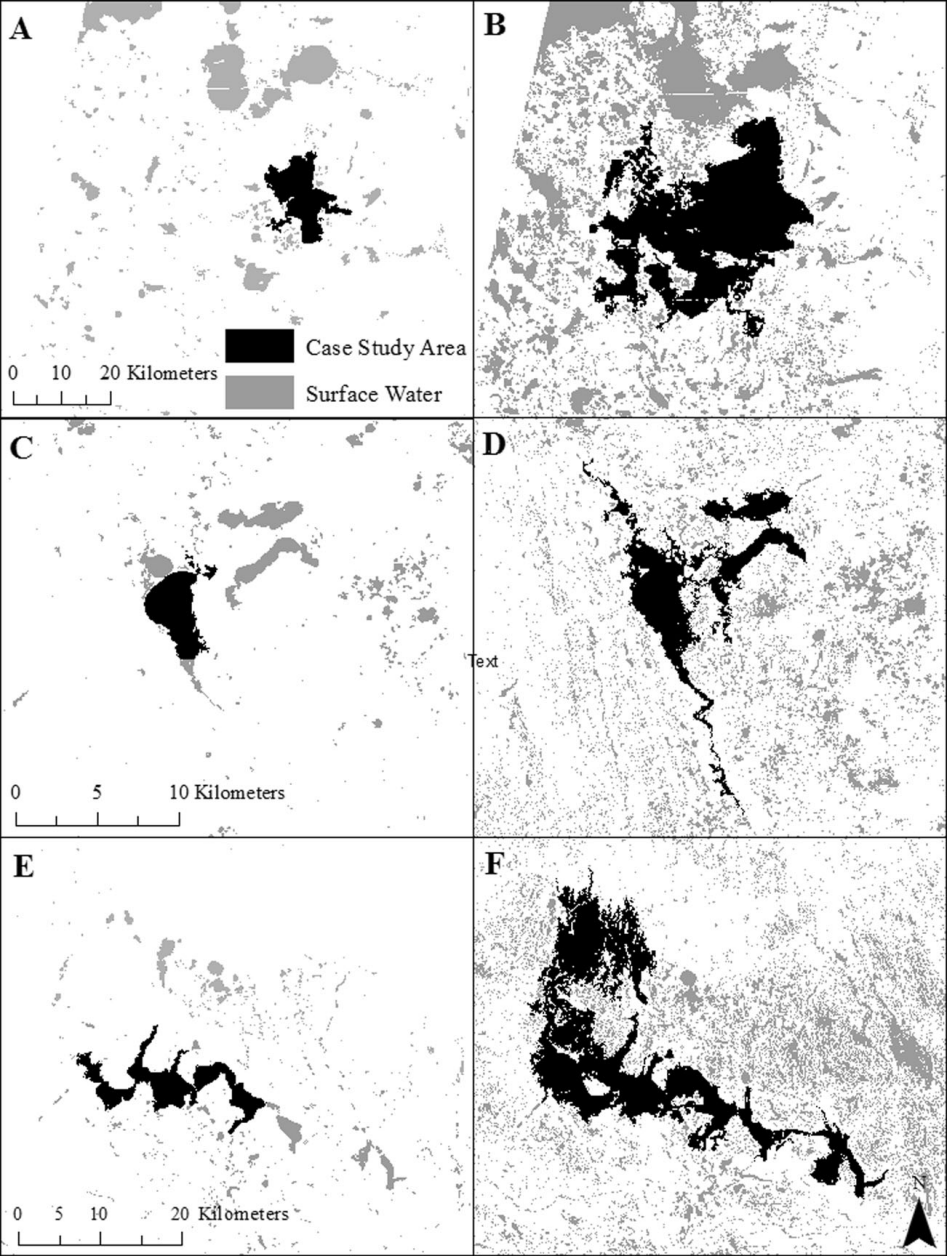
Change in lake extent from drought (spring 1990 inundated threshold) (**a**, **c**, **e**) to deluge (spring 2011 saturated threshold) (**b**, **d**, **f**) for Bitter Lake (**a** and **b**), Lake Thompson (**c** and **d**), and Devils Lake (**e** and **f**)

**Fig. 7:**
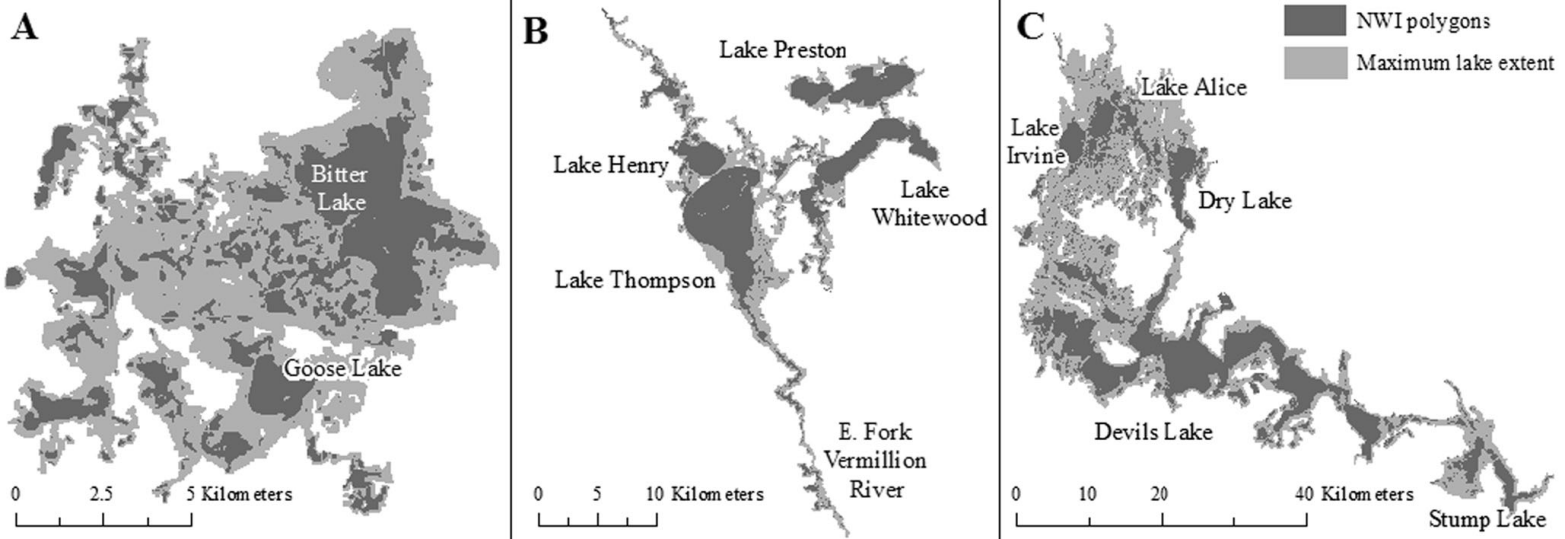
The spatial distribution of subsumed wetlands at the maximum lake extent (using the saturated threshold) of **a**) Bitter Lake, **b**) Lake Thompson, and **c**) Devils Lake

**Fig. 8:**
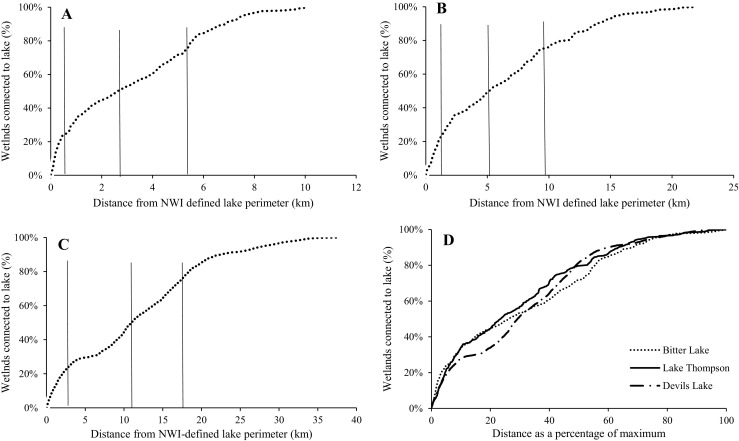
The cumulative distribution of wetlands (using the saturated threshold) showing surface-water connections with **a**) Bitter Lake, **b**) Lake Thompson, and **c**) Devils Lake, as a function of distance from the NWI-defined lake perimeter. Quartiles show the distance at which 25, 50 and 75 % of all wetlands have become part of the lake complex to (*solid vertical lines*). **d**) The normalized distribution of wetlands showing surface-water connections with each lake as a function of distance from NWI-defined lake perimeter

**Fig. 9:**
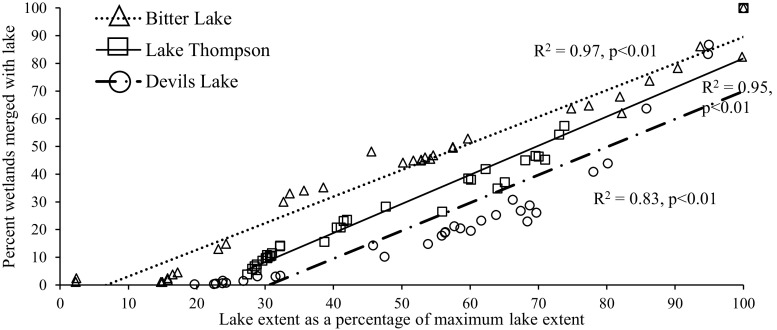
The percentage of wetlands showing surface-water connections by each lake as the Landsat lake extent expands. Both inundated and saturated values over the time series are included

For the specific large lakes examined, the extent of all three lakes increased substantially from drought to deluge conditions (Fig. 6). The rise in water level and expansion of lake extent resulted in each lake showing surface-water connections, via merging or subsuming, with a large number of wetlands under deluge conditions (Table 4). For example, at its highest water level Devils Lake showed surface-water connections with almost 6200 previously disconnected wetlands, most of which were classified by NWI as temporary (Table 4). 90 % of these wetlands were subsumed by the lake, or became part of the lake, under the lake’s maximum extent. The spatial distribution of wetlands that intersected the maximum lake extent is shown in Fig. 7. Bitter Lake, Lake Thompson, and Devils Lake showed surface-water connections with wetlands up to 10.0, 22.0 and 37.4 km, respectively, from the NWI-defined lake perimeter (Table 4), with 90 % of the wetland–lake connections occurring within 6.9, 13.9 and 22.4 km for these three lakes, respectively (Fig. 8). Despite differences in the distance over which the three lakes expanded, the percentage of wetlands connecting to or being subsumed by each lake as a function of the expanding lake extent showed similar rates of change across the three lakes (Fig. 9).

**Table 4:** Characteristics of change in lake extent and associated National Wetland Inventory (NWI) wetlands within each lake

Lake	Maximum lake extent (ha)	Total # wetlands in lake complex	Lake areal change from driest to wettest (%)	Mean (Max) distance from lake perimeter to wetland (km)	Mean size of wetlands in complex (ha)	Complex wetlands (semi-permenant, seasonal, temporary) (%)	Mean % wetlands subsumed over time series
Devils Lake	90 022	6192 (5603 subsumed, 589 merged)	408.4	11.6 (37.4)	7.1 ± 2.3	12.9, 23.2, 64.0	76.8 ± 2.0
Bitter Lake	8837	369 (318 subsumed, 51 merged)	577.7	3.1 (10.1)	9.0 ± 3.6	35.8, 27.8, 36.4	79.4 ± 5.1
Lake Thompson	16 584	442 (254 subsumed, 188 merged)	237.9	6.2 (22.0)	25.7 ± 11.0	42.6, 34.7, 22.7	68.4 ± 4.0

Wetlands connected to a lake under both the saturated and inundated thresholds were assumed to be subsumed, while wetlands connected only under the saturated threshold were assumed to have merged with lake (i.e., were not subsumed). Plus and minus standard error

## Discussion

Lake expansion can be important for understanding how surface water moves across the landscape and how surface-water connections occur between waterbody features. It is particularly relevant in landscapes with 1) low topographic gradients, 2) low infiltration rates and 3) low stream density. In such landscapes, runoff events rarely satisfy the volume threshold for surface storage in the entire basin, meaning that instead of leaving the watershed as stream discharge, excess water (i.e., precipitation inputs exceeding soil infiltration and evapotranspiration) remains as surface water, resulting in rising water levels and floods (Aragón et al. 2011; Shaw et al. 2013; Kuppel et al. 2015). Examples of landscapes that show these characteristics include low gradient floodplains (Hamilton 2002; Hamilton et al. 2004; Kuppel et al. 2015), permafrost landscapes (Smith et al. 2007), and former glacial landscapes, which are expanding globally as glaciers recede (Stokes et al. 2007; Yao et al. 2007). As a former glacial landscape, the PPR shows substantial change in surface-water extent in response to varying climate conditions (Niemuth et al. 2010; Huang et al. 2011a). Huang et al. (2011a) showed a 600 % increase in total surface-water area in the Cottonwood Lake area between 1982 and 2009. Efforts to understand how surface-water expansion can connect features across the landscape have been restricted to wetland–wetland connections (Leibowitz and Vining 2003; Kahara et al. 2009), and connections between wetlands and streams (Huang et al. 2011b; Shaw et al. 2012; Vanderhoof et al. 2015). This study extends previous findings by demonstrating that high lake water levels during wet conditions in the PPR can result in substantial surface-water expansion, connecting and subsuming wetlands previously disconnected by long distances (up to 37 km). This process is temporally dynamic, and overall wetland function on these landscapes can be expected to vary correspondingly. Wetlands merge with lakes as lake levels rise, but can retain wetland function while hydroperiod and habitat structures remain intact. As lake levels continue to rise, merged wetlands are completely subsumed by lakes and no longer function as wetlands (Mortsch 1998). Although our time series represented a general trend from dry to wet conditions, fluctuations in wetness conditions within the time series saw lakes expand and contract, connecting and disconnecting lakes, streams and wetlands multiple times over the 21-year study period.

Analysis of the three specific lakes was not meant to be representative of regional lakes but instead to demonstrate the capacity of large lakes to expand over substantial distances (>10 km), subsuming wetlands of all sizes and types. Despite site-specific differences in lake size, depth, and glacial deposits, when results were normalized the three lakes showed remarkably similar linear relationships between lake extent and the number of wetlands showing surface-water connections. Differences in the degree and persistence of lake expansion can be attributed in part to lake size, as well as to basin shape and topography of the surrounding landscape (Hayashi and van der Kamp 2000; Zhang et al. 2009; Rover et al. 2011; Shook et al. 2015). As a post-glacial landscape, topography and basin shapes across the PPR are primarily determined by the distribution and type of glacial deposits (Flint 1971; Sloan 1972). For instance, slow buildup of water-laden sediments in the lake bed of Devils Lake during the glacial period left this basin even flatter than the surrounding Drift Plains, allowing for large changes in lake extent (Wiche 1996). Presence of wetland–lake surface-water connections will also depend on the spatial distribution of wetlands in relation to lakes (Kahara et al. 2009). Both the Prairie Coteau and the Devils Lake Plain are characterized by high wetland density, a product of glacial deposits (Flint 1971; Sloan 1972; USGS 2013a), which increases the likelihood that lake expansion will subsume nearby wetlands.

For most wetlands, no surface-water connections were observed over the 21-year study period. This is due in part to limitations of existing stream datasets and the resolution of Landsat, which make it difficult to detect flowpaths that are narrow (e.g., ephemeral channels, swales, ditches) and temporary (e.g., wetland fill-and-spill in response to rain events). The aim of this analysis was to quantify lake expansions, which can merge substantial numbers of wetlands into a continuous water surface over long distances and are therefore critical to any landscape-scale analysis of surface-water connections. However, we did not assess transitions from wetlands to lake over the time series, meaning our interpretation of lake abundance and distribution is likely conservative during the wetter periods of the time series. In addition to the need to account for transitions between a waterbody functioning as a wetland versus a lake, a complete analysis of hydrological connections among water features would also need to consider narrow and temporary surface-water connections, as well as shallow and deep groundwater connections. A comprehensive analysis of watershed connectivity would also require an assessment of chemical and biological connections (U.S. EPA 2015).

Agricultural activity including row crop agriculture is common across our study area and can produce mixed effects on surface-water extent and connectivity. Ditches, pipes and field tiles can increase connectivity between waterbody features, while both filling wetlands with soil and lowering the water table through increased water withdrawal can decrease expected surface-water connectivity (De Laney 1995; Blann et al. 2009). For lakes, artificial barriers such as dams and roads can prevent or limit lake expansion, while water withdrawal can reduce lake extent. Therefore in agricultural landscapes, such as the PPR, it is challenging to separate the effects of intrinsic (climate, topography, geology) and anthropogenic drivers of variability in surface-water extent.

This research adds to our understanding of how and where surface-water interactions between wetlands, lakes and streams changes over time, and has implications for how such variation in connectivity could impact down-gradient waters. However, uncertainty remains regarding the commonality of past climate conditions as well as climate change predictions for the region. Over the past 100 years, Millett et al. (2009) have documented a significant wetting trend in the eastern portion of the PPR, where the three specific lakes considered in the analysis occur, suggesting that despite observed increases in evaporative demand (Johnson et al. 2010), the documented maximums in surface-water extent and consequent loss of wetland function due to rising lake water levels could become more common. Paleoclimate records suggest meanwhile that climate conditions over the last 100 years might not represent the full range of climate variability over the past 2000 years (Woodhouse and Overpeck 1998). The three lakes in this analysis, while extremes, provide examples of how expanding lake extent can result in the inundation of substantial numbers of previously surficially-disconnected wetlands. Our landscape-scale analysis indicates that lakes play critical roles in facilitating surface-water connections between larger numbers of wetlands per complex relative to non-lake surficially-connected complexes.

## Conclusion

The difference in surface-water extent between dry and wet periods across the PPR was substantial, and manifested as expansion of both lakes and wetlands. High lake water levels played a considerable role in facilitating surface-water connections between wetlands, as wetlands merged with and were ultimately subsumed by lakes under wet conditions. Analysis of three specific lakes showed that the expansion of lake perimeters can subsume wetlands over distances up to tens of kilometers, and that the number of wetlands showing surface-water connections with lakes may be linearly related to the distance of lake expansion. Because climate conditions across the PPR are highly variable, understanding historic patterns of surface-water expansion and contraction across the landscape will be critical to predicting long-term trends in wetland loss due to rises in surface-water level, as well as the influence of wetlands on downstream waters, under climate change conditions.
